# Singular Moser-Trudinger inequality with the exact growth condition on hyperbolic space

**DOI:** 10.1186/s13660-017-1398-8

**Published:** 2017-06-02

**Authors:** Zhao Liu, Lu Chen

**Affiliations:** 1 School of Mathematics and Computer Science, Jiangxi Science and Technology Normal University, Nanchang, 330038 China; 2 School of Mathematical Sciences, Beijing Normal University, Beijing, 100875 China

**Keywords:** 42B35, 42B37, exact growth, hyperbolic space, singular Moser-Trudinger inequality

## Abstract

In this paper, we are concerned with a singular version of the Moser-Trudinger inequality with the exact growth condition in the *n*-dimension hyperbolic space $\mathbb{H}^{n}$. Our result is a natural extension of the work of Lu and Tang in (J. Geom. Anal. 26:837-857, 2016).

## Introduction

Let $W^{1,p}_{0}(\Omega )$ denote the usual Sobolev space, i.e., the completion of $C_{0}^{\infty }(\Omega )$ under the Sobolev norm

$$ \Vert u \Vert _{W^{1,p}_{0}(\Omega )}= \biggl( \int_{\Omega }\vert \nabla u \vert ^{p}+\vert u \vert ^{p}\,dx \biggr)^{\frac{1}{p}}. $$

The classical Sobolev embedding theorem states $W^{1,n}_{0}(\Omega ) \subset L^{q}(\Omega )$ for all $1\leq q<\infty $ but $W^{1,n}_{0}( \Omega )\nsubseteq L^{\infty }(\Omega )$. One can check this by choosing the function $u(x)=\ln (\ln \frac{4R}{\vert x-x_{0} \vert })$ for some $R>0$, where $x_{0}$ is a fixed point in Ω. It is natural to ask whether there exists an optimal embedding in this limiting case. Yudovic [2], Pohozaev [3] and Trudinger [4] showed that, for some $\alpha >0$, $W^{1,n}_{0}(\Omega )$ is continuously embedded into an Orlicz space with the Young function $\phi_{\alpha }(t)=\exp (\alpha \vert t \vert ^{\frac{n}{n-1}}-1)$. Moser in [5] sharpened the exponent *α* and obtained the following result.

### Theorem A

[5]

*Let* Ω *be a bounded domain i n* $\mathbb{R} ^{n}$ ($n\geq 2$). *Then there exists a positive constant* $C_{n}$ *and a sharp constant* $\alpha_{n} =n\omega_{n-1}^{\frac{1}{n-1}}$ *such that*

$$ \frac{1}{\vert \Omega \vert } \int_{\Omega }\exp \bigl( \alpha \vert u \vert ^{\frac{n}{n-1}} \bigr)\,dx \leq C_{n} $$

*for any* $\alpha \leq \alpha_{n}$ *and* $u\in C_{0} ^{\infty }( \Omega )$ *with* $\int_{\Omega } \vert \nabla u \vert ^{n}\,dx \leq 1$, *where* $\omega_{n-1}$ *is the area of the surface of the unit ball*.

The above inequality is often referred as the Moser-Trudinger inequality. Carleson and Chang [6] employed a symmetrization and rearrangement arguments to obtain the extremals of the Moser-Trudinger inequality when Ω is a disk in $\mathbb{R}^{2}$. Later, the results of Carleson and Chang were extended by Flucher [7] to arbitrary domains in $\mathbb{R}^{2}$, and by Lin [8] for arbitrary domains in $\mathbb{R}^{n}$. The existence of extremals of the Moser-Trudinger inequality was also extended to compact Riemannian manifold cases by Li in [9, 10]. Cohn and Lu [11] were concerned with the sharp constants for the Moser-Trudinger inequality on a bounded domain on Heisenberg group $\mathbb{H}^{n}$. The singular version of the Moser-Trudinger inequality on the Heisenberg group was also proved by Lam, Lu and Tang in [12]. For more results as regards the Moser-Trudinger inequalities and applications in partial differential equations, please see [13–19] and the references therein.

A natural idea is to consider the Moser-Trudinger inequality in the whole space $\mathbb{R}^{n}$. Adachi and Tanaka in [20] proved the following nice result.

### Theorem B

[20]

*For* $0<\alpha <\alpha_{n}$, *there exists a positive constant* $C_{n}$ *such that*

$$\sup_{u\in {W^{1,n}(\mathbb{R}^{n})},\int_{\mathbb{R}^{n}}\vert \nabla u\vert ^{n}\,dx\leq 1} \int_{\mathbb{R}^{n}}\Phi \bigl(\alpha \bigl\vert u(x) \bigr\vert ^{\frac{n}{n-1}}\bigr)\,dx \leq { C_{n} \int_{\mathbb{R}^{n}}\bigl\vert u(x) \bigr\vert ^{n}\,dx,} $$

*where* $\Phi (t):=e^{t}-\sum_{i=0}^{n-2}\frac{t^{i}}{i!}$. *Moreover*, *the constant* $\alpha_{n}$ *is sharp in the sense that if* $\alpha \geq {\alpha_{n}}$, *the supremum will become infinite*.

They proved the sharpness of the exponent *α* by modifying a sequence of test functions introduced by Moser. In order to obtain the Moser-Trudinger inequality in the critical case $\alpha =\alpha_{n}$, Ruf [21] (in the dimension $n=2$) and Li and Ruf [22] (in the dimension $n\geq 3$) replaced the Dirichlet norm with the standard Sobolev norm, i.e.

$$ \Vert u \Vert _{W^{1,n}_{0}(\mathbb{R}^{n})}= \biggl( \int_{\mathbb{R}^{n}}\vert \nabla u\vert ^{n}+ \vert u \vert ^{n}\,dx \biggr)^{\frac{1}{n}}, $$

and obtained the Moser-Trudinger inequality in the whole space $\mathbb{R}^{n}$ in the case of $\alpha =\alpha_{n}$. Masmoudi and Sani in their elegant papers [23, 24] kept the two conditions $\alpha =\alpha_{n}$ and $\int_{\mathbb{R}^{n}}\vert \nabla u \vert ^{n}\,dx\leq 1$. They proved the following.

### Theorem C

[24]

*For* $n\geq 2$, *there exists a positive constant* $C_{n}$ *such that*

$$ \int_{\mathbb{R}^{n}}\frac{\Phi (\alpha_{n}\vert u \vert ^{\frac{n}{n-1}})}{(1+\vert u \vert )^{ \frac{n}{n-1}}}\,dx \leq C_{n} \int_{\mathbb{R}^{n}}\bigl\vert u(x) \bigr\vert ^{n}\,dx,\quad \forall u\in {W^{1,n} \bigl(\mathbb{R}^{n}\bigr)} \textit{ with } \int_{\mathbb{R}^{n}}\vert \nabla u \vert ^{n}\,dx\leq 1. $$

*Moreover*, *this inequality fails if the power* $\frac{n}{n-1}$ *in the denominator is replaced by any* $p<\frac{n}{n-1}$.

The above result was also extended by Chen and Liu [25] to singular version through the change of variables developed by Dong and Lu in [17].

This paper is concerned with a singular version of Moser-Trudinger inequality with the exact growth condition on hyperbolic space $\mathbb{H}^{n}$. The hyperbolic space $\mathbb{H}^{n}$ ($n\geq 2$) is a complete and simply connected Riemannian manifold and has constant curvature equal to −1. There exist many types of models for hyperbolic space $\mathbb{H}^{n}$. However, the most important models are the half-space model, the ball model, and the hyperboloid (or Lorentz) model. Throughout this paper, we are concerned about the ball model because we can use symmetry and rearrangement argument in this setting.

We define $B^{n}=\{x\in \mathbb{R}^{n} : \vert x \vert <1\}$ as a pen unit ball in $\mathbb{R}^{n}$ equipped with the Riemannian metric $g_{ij}=( \frac{1}{1-\vert x \vert ^{2}})^{2}\delta_{ij}$, which is referred to as the ball model of the hyperbolic space $\mathbb{H}^{n}$. A direct computation shows that the volume element of hyperbolic space $\mathbb{H}^{n}$ is given by $dV=(\frac{2}{1-\vert x \vert ^{2}})^{n}\,dx$ and $d(0,x)=\ln \frac{1+\vert x \vert }{1-\vert x \vert }$, where *dx* denotes the Lebesgue measure in $\mathbb{R}^{n}$ and $d(0,x)$ denotes the hyperbolic distance between the origin and *x*. It is well known that the hyperbolic gradient $\nabla_{g}$ is defined as the $\nabla_{g}=(\frac{1-\vert x \vert ^{2}}{2})^{2} \nabla $, where ∇ denotes the general gradient in $\mathbb{R} ^{n}$.

Let Ω be a domain with finite measure on hyperbolic space $\mathbb{H}^{n}$. Denote $\Vert f \Vert _{L^{n}(\Omega )}= (\int_{\Omega }\vert f \vert ^{n}\,dV )^{\frac{1}{n}}$. A straightforward calculation yields

$$ \Vert \nabla_{g} f \Vert _{L^{n}(\Omega )} = \biggl(\int_{\Omega } \langle \nabla_{g} f, \nabla_{g} f \rangle _{g}^{n/2}\,dV \biggr)^{\frac{1}{n}}= \biggl( \int_{\Omega }\vert \nabla f \vert ^{n}\,dV \biggr)^{\frac{1}{n}} $$

and

$$ \Vert \nabla_{g} f \Vert _{L^{n}(\mathbb{H}^{n})}= \biggl( \int_{\mathbb{H}^{n}} \langle \nabla_{g} f,\nabla_{g} f \rangle_{g}^{n/2}\,dV \biggr)^{\frac{1}{n}}= \biggl( \int_{B^{n}}\vert \nabla f \vert ^{n}\,dV \biggr)^{\frac{1}{n}}. $$

We also define $W^{1,n}(\mathbb{H}^{n})$ as the completion of $C_{0}^{\infty }(\mathbb{H}^{n})$ with the norm

$$ \Vert u \Vert _{W^{1,n}(\mathbb{H}^{n})}= \biggl( \int_{\mathbb{H}^{n}}\vert \nabla_{g}u\vert ^{n}+ \vert u\vert ^{n}\,dV \biggr)^{\frac{1}{n}}. $$

The Moser-Trudinger inequality on hyperbolic space was first established by Mancini and Sandeep [26], they proved the Moser-Trudiner inequality on conformal discs. Lu and Tang in [27] considered the subcritical Moser-Trudinger inequality on high dimensional hyperbolic space. They proved the following result.

### Theorem D

*For any* $u\in W^{1,n}(\mathbb{H}^{n})$ *satisfying* $\int_{\mathbb{H}^{n}}\vert \nabla_{g} u \vert ^{n}\,dV \leq 1$, *there exists a positive constant* $C_{n}$ *such that*

$$ \begin{aligned} \int_{\mathbb{H}^{n}}\frac{\Phi (\alpha (1-\frac{\beta }{n})\vert u \vert ^{ \frac{n}{n-1}})}{(d(0,x))^{\beta }}\,dV \leq C_{n} \int_{\mathbb{H}^{n}}\frac{\vert u(x) \vert ^{n}}{(d(0,x))^{\beta }}\,dV \end{aligned} $$

*for any* $\alpha <\alpha_{n}$. *Furthermore the constant* $\alpha_{n}$ *is sharp in the sense that the inequality does not hold if we replace the constant* *α* *with any* $\alpha \geq \alpha_{n}$.

Recently, Lu and Tang in [1] considered the sharp Moser-Trudinger inequality with the exact growth condition on hyperbolic space, they proved the following results.

### Theorem E

*For any* $u\in W^{1,n}(\mathbb{H}^{n})$ *satisfying* $\int_{\mathbb{H}^{n}}\vert \nabla_{g} u \vert ^{n}\,dV \leq 1$, *there exists a positive constant* $C_{n}$ *such that*

1 $$ \begin{aligned} \int_{\mathbb{H}^{n}}\frac{\Phi (\alpha_{n}\vert u \vert ^{\frac{n}{n-1}})}{(1+u)^{ \frac{n}{n-1}}}\,dV \leq C_{n} \int_{\mathbb{H}^{n}}\bigl\vert u(x) \bigr\vert ^{n}\,dV. \end{aligned} $$

*Furthermore*, *the power* $\frac{n}{n-1}$ *is sharp in the sense if the power* $\frac{n}{n-1}$ *is replaced by any* $p<\frac{n}{n-1}$, *the* (1) *become infinite*.

Motivated by the above results, we consider the singular version of the Moser-Trudinger inequality with the exact growth condition on hyperbolic space. We state our results as follows.

### Theorem 1

*For any radially decreasing function* $u\in W^{1,n}(\mathbb{H}^{n})$ *satisfying* $\int_{\mathbb{H}^{n}}\vert \nabla_{g} u \vert ^{n}\,dV \leq 1$, *there exists a positive constant* $C_{n}$ *independent of* *u* *such that*

2 $$ \begin{aligned} \int_{\mathbb{H}^{n}}\frac{\Phi (\alpha_{n}(1-\frac{\beta }{n})\vert u \vert ^{ \frac{n}{n-1}})}{(1+u)^{\frac{n}{n-1}}(d(0,x))^{\beta }}\,dV \leq C_{n} \int_{\mathbb{H}^{n}}\frac{\vert u(x) \vert ^{n}}{(d(0,x))^{\beta }}\,dV. \end{aligned} $$

We verify that the power $\frac{n}{n-1}$ is optimal.

### Theorem 2

*If the power* $\frac{n}{n-1}$ *is replaced by any* $p< \frac{n}{n-1}$, *there exists a sequence of functions* $\{u_{k}\}$ *such that* $\int_{\mathbb{H}^{n}}\vert \nabla_{g} u_{k} \vert ^{n}\,dV \leq 1$, *but*

$$ \int_{\mathbb{H}^{n}}\frac{\Phi (\alpha_{n}(1-\frac{\beta }{n})\vert u_{k} \vert ^{ \frac{n}{n-1}})}{(1+u_{k})^{p}(d(0,x))^{\beta }}\,dV \biggl( \int_{\mathbb{H}^{n}}\frac{\vert u_{k}(x) \vert ^{n}}{(d(0,x))^{\beta }}\,dV \biggr)^{-1}\rightarrow \infty . $$

This paper is organized as follows. In Section 2, we give some important lemmas which will play key roles in the proof of Theorem 1. In Section 3, we establish a singular version of Moser-Trudinger inequality with the exact growth condition on hyperbolic space (Theorem 1). In Section 4, we give the proof of the sharpness of the singular Moser-Trudinger inequality with the exact growth condition in Theorem 1.

## Some important lemmas

In this section, we give some key lemmas which play an important role in the proof of Theorem 1.

### Lemma 3

see [1, 23]

*Given any sequence* $a=\{a_{k}\}_{k\geq 1}$, *let* $\Vert a \Vert _{1}=\sum_{k=0}^{+\infty }\vert a_{k} \vert $, $\Vert a \Vert _{n}=(\sum_{k=0}^{+ \infty }\vert a_{k} \vert ^{n})^{1/n}$, $\Vert a \Vert _{(e)}=(\sum_{k=0}^{+\infty }\vert a_{k} \vert ^{n}e^{k(1-\frac{\beta }{n})})^{1/n}$ *and* $\mu (h)=\inf \{\Vert a \Vert _{(e)}:\Vert a \Vert _{1}=h, \Vert a \Vert _{n}\leq 1\}$. *Then*, *for any* $h>1$, *we have*

$$ \mu^{n}(h)\sim \frac{e^{(1-\frac{\beta }{n})h^{\frac{n}{n-1}}}}{h^{ \frac{n}{n-1}}}. $$

With the help of Lemma 3, one can obtain the following lemma.

### Lemma 4

*There exists a constant* *C* *such that for*, *any nonnegative decreasing function* *u* *with* $u(R)>K^{\frac{1}{n}}$ *and* $\omega_{n-1}\int_{R} ^{\infty } \vert u' \vert ^{n}t^{n-1}\,dt\leq K$ *for some* $R, K>0$,

$$ \frac{e^{\alpha_{n}(1-\frac{\beta }{n})K^{-\frac{1}{n-1}}u^{ \frac{n}{n-1}}(R)}}{u^{\frac{n}{n-1}}(R)}R^{n-\beta } \leq C\frac{\int _{R}^{\infty }u^{n}t^{n-\beta -1}\,dt}{K^{\frac{n}{n-1}}}. $$

### Proof

By scaling, it suffices to show that, for any nonnegative decreasing function *u* satisfying $u(1)>1$ and $\omega_{n-1}\int_{1}^{\infty }\vert u' \vert ^{n}t ^{n-1}\,dt\leq 1$,

$$ \frac{e^{\alpha_{n}(1-\frac{\beta }{n})u^{\frac{n}{n-1}}(1)}}{u^{ \frac{n}{n-1}}(1)}\lesssim \int_{1}^{\infty }u^{n}t^{n-\alpha -1}\,dt. $$

Let $h_{k}=\alpha_{n}^{\frac{n-1}{n}}u(e^{k/n})$, $a_{k}=h_{k}-h_{k+1}$ and $a=\{a_{k}\}$. Then $a_{k}\geq 0$ and

$$ {\sum_{k\geq 0}\vert a_{k} \vert =h_{0}=\alpha_{n}^{\frac{n-1}{n}}u(1}). $$

Since

$$ \begin{aligned} h_{k}-h_{k+1} &= \alpha_{n}^{\frac{n-1}{n}} \bigl(u\bigl(e^{\frac{k}{n}}\bigr)-u\bigl(e ^{\frac{k+1}{n}}\bigr) \bigr) \\ &=\alpha_{n}^{\frac{n-1}{n}} \int_{e^{\frac{k+1}{n}}}^{e^{\frac{k}{n}}}u'(t)\,dt \\ &=\alpha_{n}^{\frac{n-1}{n}} \biggl( \int_{e^{\frac{k}{n}}}^{e^{ \frac{k+1}{n}}}\bigl\vert u'(t) \bigr\vert ^{n}t^{n-1}\,dt \biggr)^{\frac{1}{n}} \biggl( \int_{e^{\frac{k}{n}}}^{e^{\frac{k+1}{n}}}\frac{1}{t}\,dt \biggr)^{ \frac{n-1}{n}} \\ &= \biggl(\omega_{n-1} \int_{1}^{\infty }\bigl\vert u'(t) \bigr\vert ^{n}t^{n-1}\,dt \biggr)^{ \frac{1}{n}}, \end{aligned} $$

we have

$$ \begin{aligned} \Vert a \Vert _{n}&= \biggl(\sum_{k\geq 0} \vert a_{k} \vert ^{n} \biggr)^{1/n} \\ &= \biggl( \sum_{k\geq 0}\vert h_{k}-h_{k+1} \vert ^{n} \biggr)^{1/n}\leq 1. \end{aligned} $$

Moreover,

$$ \begin{aligned} \int_{1}^{\infty }u^{n}t^{n-\beta -1}\,dt &=\sum _{k\geq 0} \int_{e^{\frac{k}{n}}}^{e^{\frac{k+1}{n}}}u^{n}t^{n-\beta -1}\,dt \\ &=\sum_{k\geq 0}\bigl(u\bigl(e^{\frac{k+1}{n}}\bigr) \bigr)^{n} \int_{e^{ \frac{k}{n}}}^{e^{\frac{k+1}{n}}}t^{n-\beta -1}\,dt \\ &\gtrsim \sum_{k\geq 0}\bigl(u\bigl(e^{\frac{k+1}{n}} \bigr)\bigr)^{n}e^{(1-\frac{ \beta }{n})(k+1)} \\ &=\sum_{k\geq 1}\bigl(u\bigl(e^{\frac{k}{n}}\bigr) \bigr)^{n}e^{(1- \frac{\beta }{n})k} \\ &\gtrsim \sum_{k\geq 1}h_{k}^{n}e^{(1-\frac{\beta }{n})k} \\ &\gtrsim \sum_{k\geq 1}a_{k}^{n}e^{(1-\frac{\beta }{n})k}. \end{aligned} $$

Therefore,

3 $$\begin{aligned} \Vert a \Vert _{(e)} &=a_{0}^{n}+\sum_{k\geq 1}a_{k}^{n}e^{(1-\frac{ \beta }{n})k} \\ &\leq h_{0}^{n}+\sum_{k\geq 1}a_{k}^{n}e^{(1-\frac{\beta }{n})k} \\ &\lesssim h_{0}^{n}+ \int_{1}^{\infty }u^{n}t^{n-\beta -1}\,dt. \end{aligned}$$

Next, we start to estimate $h_{0}$. Set $1< r< e^{1/{4n}}$, then

$$ \begin{aligned} h_{0}-\alpha_{n}^{\frac{n-1}{n}}u(r) &=\alpha_{n}^{\frac{n-1}{n}} \int _{1}^{r}\bigl\vert u'(t) \bigr\vert \,dt \\ &=\alpha_{n}^{\frac{n-1}{n}} \biggl( \int_{1}^{r}\bigl\vert u'(t) \bigr\vert ^{n}t^{n-1}\,dt \biggr)^{\frac{1}{n}} \biggl( \int_{1}^{r}\frac{1}{t}\,dt \biggr)^{ \frac{n-1}{n}} \\ &=4^{-\frac{n-1}{n}} \biggl(\omega_{n-1} \int_{1}^{r}\bigl\vert u'(t) \bigr\vert ^{n}t^{n-1}\,dt \biggr)^{\frac{1}{n}} \\ &\leq \frac{h_{0}}{2} \end{aligned} $$

and

4 $$ \int_{1}^{\infty }u^{n}t^{n-\beta -1}\,dt\geq \int_{1}^{e^{1/{4n}}}u ^{n}t^{n-\beta -1}\,dt \gtrsim h_{0}^{n}. $$

By (3) and (4), we derive that

$$ \Vert a \Vert _{(e)}\lesssim \int_{1}^{\infty }u^{n}t^{n-\beta -1}\,dt. $$

Then we apply Lemma 3 to conclude that

$$ \begin{aligned} \int_{1}^{\infty }u^{n}t^{n-\beta -1}\,dt&\gtrsim \frac{e^{(1-\frac{ \beta }{n})h_{0}^{\frac{n}{n-1}}}}{h_{0}^{\frac{n}{n-1}}} \\ &=\frac{e ^{\alpha_{n}(1-\frac{\beta }{n})u^{\frac{n}{n-1}}(1)}}{\alpha_{n}u ^{\frac{n}{n-1}}(1)}. \end{aligned} $$

This completes the proof of Lemma 4. □

## Singular Moser-Trudinger inequality with the exact growth condition

In this section, we shall establish a singular version of Moser-Trudinger inequality with the exact growth condition on hyperbolic space. Namely, we will give the proof of Theorem 1. By the density, we can assume that $u(x)$ is compactly supported in $\mathbb{H}^{n}$. We use the idea of Moser [5]. Set $d(0,x)=t$ and $u(x)=v(d(0,x))=v(t)$, then

$$\begin{aligned} &\int_{\mathbb{H}^{n}}\frac{\Phi (\alpha_{n}(1-\frac{\beta }{n})\vert u \vert ^{ \frac{n}{n-1}})}{(1+u)^{\frac{n}{n-1}}(d(0,x))^{\beta }}\,dV= \omega _{n-1} \int_{0}^{\infty }\frac{\Phi (\alpha_{n}(1-\frac{\beta }{n})\vert v \vert ^{ \frac{n}{n-1}})}{(1+v)^{\frac{n}{n-1}}} t^{-\beta }( \sinh t)^{n-1}\,dt, \\ &\int_{\mathbb{H}^{n}}\bigl\vert \nabla_{g} u(x) \bigr\vert ^{n}\,dV=\omega_{n-1} \int_{0}^{ \infty }\bigl\vert v'(t) \bigr\vert ^{n}(\sinh t)^{n-1}\,dt, \\ &\int_{\mathbb{H}^{n}}\frac{\vert u(x) \vert ^{n}}{(d(0,x))^{\beta }}\,dV=\omega _{n-1} \int_{0}^{\infty }\bigl\vert v(t) \bigr\vert ^{n}(\sinh t)^{n-1}t^{-\beta }\,dt. \end{aligned}$$

Thus, it suffices to show that there exists a positive constant $C_{n}$ such that

$$ \int_{0}^{\infty }\frac{\Phi (\alpha_{n}(1-\frac{\beta }{n})\vert v \vert ^{ \frac{n}{n-1}})}{(1+v)^{\frac{n}{n-1}}} t^{-\beta }( \sinh t)^{n-1}\,dt \leq C_{n} \int_{0}^{\infty }\bigl\vert v(t) \bigr\vert ^{n}(\sinh t)^{n-1}t^{-\beta }\,dt $$

for any $v(t)$ satisfying $v(t)\geq 0, v'(t)\leq 0, v(t_{0})=0$ for some $t_{0}\in \mathbb{R}$ and

$$ \omega_{n-1} \int_{0}^{\infty }\bigl\vert v'(t) \bigr\vert ^{n}(\sinh t)^{n-1}\,dt\leq 1. $$

Set $R=\sup \{t\in \mathbb{R}: v(t)\geq 1\}$. We can use $v(t)\in (0,1)$ for $t\in (R, \infty )$ to obtain

$$ \Phi \biggl(\alpha_{n}\biggl(1-\frac{\beta }{n}\biggr)\vert v \vert ^{\frac{n}{n-1}}\biggr)\leq C_{n} \bigl\vert v(t) \bigr\vert ^{n} $$

for any $t\geq R$. It follows that

5 $$\begin{aligned} & \int_{R}^{\infty }\frac{\Phi (\alpha_{n}(1-\frac{\beta }{n})\vert v \vert ^{ \frac{n}{n-1}})}{(1+v)^{\frac{n}{n-1}}} t^{-\beta }( \sinh t)^{n-1}\,dt \\ &\quad \leq \int_{R}^{\infty }\Phi \biggl(\alpha_{n} \biggl(1-\frac{\beta }{n}\biggr)\vert v \vert ^{ \frac{n}{n-1}}\biggr) t^{-\beta }(\sinh t)^{n-1}\,dt \\ & \quad \leq C_{n} \int_{R}^{\infty } \bigl\vert v(t) \bigr\vert ^{n}t^{-\beta }(\sinh t)^{n-1}\,dt. \end{aligned}$$

Next, we focus on the integral over $(0, R]$. Set $0<\varepsilon_{0}<1$ and let $R_{1}(u)>0$ such that

$$\begin{aligned}& \omega_{n-1}\int_{0}^{R_{1}}\bigl\vert v(t) \bigr\vert ^{n}(\sinh t)^{n-1}\,dt\leq \rho \varepsilon_{0} \end{aligned}$$

and

$$\begin{aligned}& \omega_{n-1}\int_{R_{1}}^{\infty }\bigl\vert v(t) \bigr\vert ^{n}( \sinh t)^{n-1}\,dt\leq \rho (1-\varepsilon_{0}), \end{aligned}$$

where $0<\rho \leq 1$.

In order to estimate the integral over $(0, R]$, we need to consider two cases: $R_{1}\geq R$ and $R_{1}\leq R$.

First, we consider the case that $R_{1}\geq R$. For $0< t\leq R$, we can write

$$ \begin{aligned} v(t) &=v(R)+ \int_{R}^{t}v'(s)\,ds \\ &\leq v(R)+ \biggl( \int_{t}^{R}\bigl\vert v'(s) \bigr\vert ^{n} (\sinh s)^{n-1}\,ds \biggr)^{ \frac{1}{n}} \biggl( \int_{t}^{R}\frac{1}{\sinh s}\,ds \biggr)^{\frac{n-1}{n}} \\ &\leq 1+ \biggl(\frac{\rho \varepsilon_{0}}{w_{n-1}} \biggr)^{\frac{1}{n}} \biggl(\ln \biggl( \frac{e^{R}-1}{e^{R}+1} \frac{e^{t}+1}{e^{t}-1} \biggr) \biggr)^{\frac{n-1}{n}}. \end{aligned} $$

For any $\varepsilon >0$, one can apply the following well-known inequality:

6 $$ 1+s^{\frac{n-1}{n}}\leq \bigl((1+\varepsilon )s+C_{\varepsilon } \bigr)^{ \frac{n-1}{n}} $$

to obtain

$$ \bigl\vert v(t) \bigr\vert ^{\frac{n}{n-1}}\leq (1+\varepsilon ) \biggl( \frac{\rho \varepsilon _{0}}{w_{n-1}} \biggr)^{\frac{1}{n-1}} \biggl(\ln \biggl(\frac{e^{R}-1}{e^{R}+1} \frac{e ^{t}+1}{e^{t}-1}\biggr) \biggr)+C_{\varepsilon }. $$

Pick *ε* sufficiently small such that $(1+\varepsilon )^{n-1} \varepsilon_{0}< 1$, then

$$ \bigl\vert v(t) \bigr\vert ^{\frac{n}{n-1}}\leq \biggl(\frac{\rho (1+\varepsilon )^{n-1} \varepsilon_{0}}{w_{n-1}} \biggr)^{\frac{1}{n-1}} \biggl(\ln \biggl(\frac{e^{R}-1}{e ^{R}+1} \frac{e^{t}+1}{e^{t}-1} \biggr) \biggr)+C_{\varepsilon_{0}}. $$

Denote $c_{0}=(n-\beta )(\rho (1+\varepsilon )^{n-1}\varepsilon_{0})^{ \frac{1}{n-1}}$. It follows that $0< c_{0}< n-\beta $ and

$$ \begin{aligned} & \int_{0}^{R}\frac{\Phi (\alpha_{n}(1-\frac{\beta }{n})\vert v \vert ^{ \frac{n}{n-1}})}{(1+v)^{\frac{n}{n-1}}} t^{-\beta }( \sinh t)^{n-1}\,dt \\ & \quad \leq \int_{0}^{R}\Phi \biggl(\alpha_{n} \biggl(1-\frac{\beta }{n}\biggr)\vert v \vert ^{ \frac{n}{n-1}}\biggr) t^{-\beta }(\sinh t)^{n-1}\,dt \\ & \quad \leq \int_{0}^{R}\Phi \biggl(\alpha_{n} \biggl(1-\frac{\beta }{n}\biggr) \biggl(\biggl(\frac{ \rho (1+\varepsilon )^{n-1}\varepsilon_{0}}{w_{n-1}} \biggr)^{\frac{1}{n-1}} \ln \biggl(\frac{e^{R}-1}{e^{R}+1} \frac{e^{t}+1}{e^{t}-1} \biggr)+C_{\varepsilon _{0}} \biggr)\biggr) \\ &\quad \quad {}\times t^{-\beta }(\sinh t)^{n-1}\,dt \\ & \quad \leq \exp \biggl(\alpha_{n}\biggl(1-\frac{\beta }{n} \biggr)C_{\varepsilon_{0}}\biggr) \biggl(\frac{e ^{R}-1}{e^{R}+1} \biggr)^{c_{0}} \int_{0}^{R}\frac{(e^{t}+1)^{c_{0}+n-1}}{(e ^{t}-1)^{c_{0}-n+1}}\frac{t^{-\beta }}{(2e^{t})^{n-1}}\,dt. \end{aligned} $$

For $R\rightarrow 0$, it is easy to check that

$$ \exp \biggl(\alpha_{n}\biggl(1-\frac{\beta }{n} \biggr)C_{\varepsilon_{0}}\biggr) \biggl(\frac{e^{R}-1}{e^{R}+1} \biggr)^{c_{0}} \int_{0}^{R}\frac{(e^{t}+1)^{c _{0}+n-1}}{(e^{t}-1)^{c_{0}-n+1}}\frac{t^{-\beta }}{(2e^{t})^{n-1}}\,dt \sim R^{n-\beta }. $$

For $R\rightarrow +\infty $, one can calculate

$$ \begin{aligned} &\exp \biggl(\alpha_{n}\biggl(1-\frac{\beta }{n}\biggr)C_{\varepsilon_{0}} \biggr) \biggl(\frac{e ^{R}-1}{e^{R}+1} \biggr)^{c_{0}} \int_{0}^{R}\frac{(e^{t}+1)^{c_{0}+n-1}}{(e ^{t}-1)^{c_{0}-n+1}}\frac{t^{-\beta }}{(2e^{t})^{n-1}}\,dt \\ &\quad {}\sim \int_{0} ^{R}\frac{(e^{t}+1)^{c_{0}+n-1}}{(e^{t}-1)^{c_{0}-n+1}} \frac{t^{- \beta }}{(2e^{t})^{n-1}}\,dt. \end{aligned} $$

On the other hand,

$$ \int_{0}^{R}\bigl\vert v(t) \bigr\vert ^{n}(\sinh t)^{n-1}t^{-\beta }\,dt\geq \int_{0}^{R}( \sinh t)^{n-1}t^{-\beta }\,dt. $$

Therefore, for $R\rightarrow 0$,

$$ \int_{0}^{R}(\sinh t)^{n-1}t^{-\beta }\,dt \sim R^{n-\beta }. $$

Thus,

7 $$\begin{aligned} &\lim_{R\rightarrow 0} \frac{\int_{0}^{R}\vert v(t) \vert ^{n}(\sinh t)^{n-1}t ^{-\beta }\,dt}{\int_{0}^{R}\frac{\Phi (\alpha_{n}(1-\frac{\beta }{n})\vert v \vert ^{ \frac{n}{n-1}})}{(1+v)^{\frac{n}{n-1}}} t^{-\beta }(\sinh t)^{n-1}\,dt} \\ & \quad \geq \lim_{R\rightarrow 0}\frac{\int_{0}^{R}(\sinh t)^{n-1}t^{-\beta }\,dt}{\exp (\alpha_{n}(1-\frac{\beta }{n})C_{\varepsilon_{0}})(\frac{e ^{R}-1}{e^{R}+1})^{c_{0}} \int_{0}^{R}\frac{(e^{t}+1)^{c_{0}+n-1}}{(e ^{t}-1)^{c_{0}-n+1}}\frac{t^{-\beta }}{(2e^{t})^{n-1}}\,dt} \\ & \quad \geq C_{0} \end{aligned}$$

and

8 $$\begin{aligned} &\lim_{R\rightarrow +\infty } \frac{\int_{0}^{R}\vert v(t) \vert ^{n}(\sinh t)^{n-1}t ^{-\beta }\,dt}{\int_{0}^{R}\frac{\Phi (\alpha_{n}(1-\frac{\beta }{n})\vert v \vert ^{ \frac{n}{n-1}})}{(1+v)^{\frac{n}{n-1}}} t^{-\beta }(\sinh t)^{n-1}\,dt} \\ & \quad \geq \lim_{R\rightarrow +\infty }\frac{\int_{0}^{R}(\sinh t)^{n-1}t ^{-\beta }\,dt}{\exp (\alpha_{n}(1-\frac{\beta }{n})C_{\varepsilon_{0}})(\frac{e ^{R}-1}{e^{R}+1})^{c_{0}} \int_{0}^{R}\frac{(e^{t}+1)^{c_{0}+n-1}}{(e ^{t}-1)^{c_{0}-n+1}}\frac{t^{-\beta }}{(2e^{t})^{n-1}}\,dt} \\ & \quad \geq \lim_{R\rightarrow +\infty }\frac{(\sinh R)^{n-1}R^{-\beta }}{ \exp (\alpha_{n}(1-\frac{\beta }{n})C_{\varepsilon_{0}})R^{-\beta }(e ^{R})^{2n-2}(2e^{R})^{1-n}} \\ &\quad = C_{1}. \end{aligned}$$

We can combine (7) and (8) to derive that

9 $$\begin{aligned} &\int_{0}^{R}\frac{\Phi (\alpha_{n}(1-\frac{\beta }{n})\vert v \vert ^{ \frac{n}{n-1}})}{(1+v)^{\frac{n}{n-1}}} t^{-\beta }( \sinh t)^{n-1}\,dt \\ &\quad \leq C_{n} \int_{0}^{R}\bigl\vert v(t) \bigr\vert ^{n}(\sinh t)^{n-1}t^{-\beta }\,dt. \end{aligned}$$

Then by (5) and (9), we obtain the desired inequality of Theorem 1 for $R_{1}\geq R$.

Now, we consider the case $R_{1}< R$. First, we consider the integral over $(R_{1}, R)$. By $\omega_{n-1}\int_{R_{1}}^{\infty }\vert v(t) \vert ^{n}( \sinh t)^{n-1}\,dt\leq \rho \varepsilon_{0}$, we have

$$ \begin{aligned} v(t) &=v(R)+ \int_{R}^{t}v'(s)\,ds \\ &\leq v(R)+ \biggl( \int_{R_{1}}^{\infty }\bigl\vert v'(s) \bigr\vert ^{n} (\sinh s)^{n-1}\,ds \biggr)^{\frac{1}{n}} \biggl( \int_{t}^{R}\frac{1}{\sinh s}\,ds \biggr)^{ \frac{n-1}{n}} \\ &\leq 1+ \biggl(\frac{\rho \varepsilon_{0}}{w_{n-1}} \biggr)^{\frac{1}{n}} \biggl(\ln \biggl( \frac{e^{R}-1}{e^{R}+1} \frac{e^{t}+1}{e^{t}-1}\biggr) \biggr)^{ \frac{n-1}{n}}, \end{aligned} $$

where $R_{1}< t< R$. Set $\varepsilon =(1+\varepsilon_{0})^{ \frac{1}{n-1}}-1$. It is easy to check that

$$ \bigl\vert v(t) \bigr\vert ^{\frac{n}{n-1}}\leq \biggl(\frac{\rho (1-\varepsilon_{0}^{2})}{w _{n-1}} \biggr)^{\frac{1}{n-1}} \biggl(\ln \biggl(\frac{e^{R}-1}{e^{R}+1} \frac{e ^{t}+1}{e^{t}-1} \biggr) \biggr)+C_{\varepsilon_{0}} $$

by (6). Denote $c_{1}=(n-\beta )(\rho (1-\varepsilon _{0}^{2}))^{\frac{1}{n-1}}$, then $0< c_{1}< n-\beta $ and

$$\begin{aligned} &\int_{R_{1}}^{R}\frac{\Phi (\alpha_{n}(1-\frac{\beta }{n})\vert v \vert ^{ \frac{n}{n-1}})}{(1+v)^{\frac{n}{n-1}}} t^{-\beta }( \sinh t)^{n-1}\,dt \\ & \quad \leq \int_{R_{1}}^{R}\Phi \biggl(\alpha_{n} \biggl(1-\frac{\beta }{n}\biggr)\vert v \vert ^{ \frac{n}{n-1}}\biggr) t^{-\beta }(\sinh t)^{n-1}\,dt \\ & \quad \leq \int_{0}^{R}\Phi \biggl(\alpha_{n} \biggl(1-\frac{\beta }{n}\biggr) \biggl(\frac{\rho (1-\varepsilon_{0}^{2})}{w_{n-1}} \biggr)^{\frac{1}{n-1}}\biggl( \ln \biggl(\frac{e^{R}-1}{e^{R}+1} \frac{e^{t}+1}{e^{t}-1}\biggr) \biggr)+C_{\varepsilon_{0}}\biggr) t^{-\beta }(\sinh t)^{n-1}\,dt \\ & \quad \leq \exp \biggl(\alpha_{n}\biggl(1-\frac{\beta }{n}\biggr)C_{\varepsilon_{0}}\biggr) \biggl(\frac{e^{R}-1}{e^{R}+1} \biggr)^{c_{1}} \int_{0}^{R}\frac{(e^{t}+1)^{c_{1}+n-1}}{(e^{t}-1)^{c_{1}-n+1}}\frac{t^{-\beta }}{(2e^{t})^{n-1}}\,dt. \end{aligned}$$

Using the same calculation as we did in the case $R_{1}\geq R$, we can derive

10 $$ \begin{aligned} \int_{R_{1}}^{R}\frac{\Phi (\alpha_{n}(1-\frac{\beta }{n})\vert v \vert ^{ \frac{n}{n-1}})}{(1+v)^{\frac{n}{n-1}}} t^{-\beta }( \sinh t)^{n-1}\,dt \leq C_{n} \int_{0}^{R}\bigl\vert v(t) \bigr\vert ^{n}(\sinh t)^{n-1}t^{-\beta }\,dt. \end{aligned} $$

Now, we only need to consider the integral on $[0, R_{1})$. Set $w(t)=v(t)-v(R_{1})$, then

$$ \omega_{n-1} \int_{0}^{R_{1}}\bigl\vert w'(t) \bigr\vert ^{n}(\sinh t)^{n-1}\,dt\leq \rho \varepsilon_{0}. $$

One can employ the equality (6) to derive that

$$ \bigl\vert v(t) \bigr\vert ^{\frac{n}{n-1}}=(1+\varepsilon )w(t)^{\frac{n}{n-1}}+C_{\varepsilon }v(R_{1})^{\frac{n}{n-1}}. $$

Then we obtain

11 $$\begin{aligned} &\int_{0}^{R_{1}}\frac{\Phi (\alpha_{n}(1-\frac{\beta }{n})\vert v \vert ^{ \frac{n}{n-1}})}{(1+v)^{\frac{n}{n-1}}} t^{-\beta }( \sinh t)^{n-1}\,dt \\ &\quad \leq \int_{0}^{R_{1}}\frac{\Phi (\alpha_{n}(1-\frac{\beta }{n})\vert v \vert ^{ \frac{n}{n-1}})}{(v(R_{1}))^{\frac{n}{n-1}}} t^{-\beta }( \sinh t)^{n-1}\,dt \\ & \quad \leq \frac{\exp (\alpha_{n}(1-\frac{\beta }{n})C_{\varepsilon }v(R _{1})^{\frac{n}{n-1}})}{(v(R_{1}))^{\frac{n}{n-1}}} \\ &\quad \quad {}\times \int_{0}^{R_{1}} \exp \biggl(\alpha_{n} \biggl(1-\frac{\beta }{n}\biggr) (1+\varepsilon ) w^{\frac{n}{n-1}}\biggr)t ^{-\beta }(\sinh t)^{n-1}\,dt. \end{aligned}$$

Set $\varepsilon =\varepsilon_{0}^{\frac{1}{1-n}}-1$, then

$$ C_{\varepsilon }=\biggl(1-\frac{1}{(1+\varepsilon )^{n-1}}\biggr)^{\frac{1}{1-n}}=(1- \varepsilon_{0})^{\frac{1}{1-n}}. $$

Since

$$ \omega_{n-1} \int_{R_{1}}^{\infty }\bigl\vert v'(t) \bigr\vert ^{n}(\sinh t)^{n-1}\,dt\leq \rho (1-\varepsilon_{0}) \leq 1-\varepsilon_{0}. $$

We can apply Lemma 4 to obtain

12 $$ \begin{aligned} \frac{\exp (\alpha_{n}(1-\frac{\beta }{n})C_{\varepsilon }v(R_{1})^{ \frac{n}{n-1}})}{(v(R_{1}))^{\frac{n}{n-1}}} \leq C_{\varepsilon_{0}} \frac{\int_{R_{1}}^{\infty } \vert v \vert ^{n} t^{n-1}\,dt}{(1-\varepsilon_{0})^{ \frac{n}{n-1}}(R_{1})^{n-\beta }}. \end{aligned} $$

Let $\Omega =\{x: d(0,x)< R_{1}\}$ and $v_{1}(x)=(1+\varepsilon )^{ \frac{n-1}{n}}w(d(0,x))$ in Ω, then

$$ \int_{\mathbb{H}^{n}}\vert \nabla_{g} v_{1} \vert ^{n}\,dV=\omega_{n-1} \int_{0} ^{R_{1}}(1+\varepsilon )^{n-1}\bigl\vert w'(t) \bigr\vert ^{n}(\sinh t)^{n-1}\,dt \leq \rho \leq 1. $$

We can apply the singular Moser-Trudinger inequality on a bounded domain to obtain

13 $$ \begin{aligned} \int_{\Omega } \exp \biggl(\alpha_{n}\biggl(1- \frac{\beta }{n}\biggr)\vert v_{1} \vert ^{ \frac{n}{n-1}}\biggr) \bigl(d(0,x)\bigr)^{-\beta }\,dV \leq C \int_{\Omega }\bigl(d(0,x)\bigr)^{- \beta }\,dV. \end{aligned} $$

That is,

$$ \int_{0}^{R_{1}} \exp \biggl(\alpha_{n} \biggl(1-\frac{\beta }{n}\biggr) (1+\varepsilon )\vert w \vert ^{ \frac{n}{n-1}}\biggr)t^{-\beta }(\sinh t)^{n-1}\,dt \leq C \int_{0}^{R_{1}}t ^{-\beta }(\sinh t)^{n-1}\,dt. $$

Since $\frac{\sinh t}{t}$ is monotone increasing on $(0, \infty )$, by (11), (12) and (13) we derive

14 $$\begin{aligned} &\int_{0}^{R_{1}}\frac{\Phi (\alpha_{n}(1-\frac{\beta }{n})\vert v \vert ^{ \frac{n}{n-1}})}{(1+v)^{\frac{n}{n-1}}} t^{-\beta }( \sinh t)^{n-1}\,dt \\ &\quad \leq C_{\varepsilon_{0}} \frac{\int_{R_{1}}^{\infty } \vert v \vert ^{n} t^{n-1- \beta }\,dt}{(R_{1})^{n-\beta }} \int_{0}^{R_{1}} t^{-\beta }(\sinh t)^{n-1}\,dt \\ & \quad \leq C_{\varepsilon_{0}} \frac{\int_{R_{1}}^{\infty } \vert v \vert ^{n} (\sinh t)^{n-1}(\frac{t}{\sinh t})^{n-1}t^{-\beta }\,dt}{(R_{1})^{n-\beta }} \int_{0}^{R_{1}} t^{n-1-\beta }\biggl( \frac{\sinh t}{t}\biggr)^{n-1}\,dt \\ &\quad \leq C_{\varepsilon_{0}} \int_{R_{1}}^{\infty } \vert v \vert ^{n} (\sinh t)^{n-1}t ^{-\beta }\,dt. \end{aligned}$$

Combining (10) with (14), we obtain the desired inequality of Theorem 1 for $R_{1}\leq R$. This accomplishes the proof of Theorem 1.

## Sharpness

In this section, we show that the desired inequality in Theorem 1 does not hold if the power $\frac{n}{n-1}$ is replaced by any $p<\frac{n}{n-1}$.

We choose $\{u_{k}\}_{k=1}^{\infty }$ as follows:

$$\begin{aligned} u_{k}(x)=\omega^{-\frac{1}{n}}_{n-1}C_{k} \textstyle\begin{cases} k^{\frac{n-1}{n}},& \text{if } 0\leq d(0,x)\leq e^{-k}, \\ \vert \ln d(0,x) \vert k^{-\frac{1}{n}},& \text{if } e^{-k}< d(0,x)\leq 1, \\ 0,& \text{if } d(0,x)>1, \end{cases}\displaystyle \end{aligned}$$

where $C_{k}=(k^{-1}\int_{e^{-k}}^{1} t^{-n}(\sinh t)^{n-1}\,dt)^{- \frac{1}{n}}$. It is easy to check that $C_{k} \rightarrow 1$ and $C_{k}^{\frac{n}{n-1}}k-k\rightarrow 0$ as $k\rightarrow \infty $. Let $v_{k}(d(0,x))=u_{k}(x)$, then

$$\begin{aligned} v_{k}(t)=\omega^{-\frac{1}{n}}_{n-1}C_{k} \textstyle\begin{cases} k^{\frac{n-1}{n}},& \text{if } 0\leq t\leq e^{-k}, \\ \vert \ln t \vert k^{-\frac{1}{n}},& \text{if } e^{-k}< t\leq 1, \\ 0,& \text{if } t>1. \end{cases}\displaystyle \end{aligned}$$

By calculation, we derive that

$$ \begin{aligned} \int_{\mathbb{H}^{n}}\vert \nabla_{g} u_{k} \vert ^{n}\,dV &=\omega_{n-1} \int_{0} ^{\infty }\bigl\vert v_{k}(t) \bigr\vert ^{n}(\sinh t)^{n-1}\,dt \\ &=\frac{(C_{k})^{n}}{k} \int_{e^{-k}}^{1}t^{-n}(\sinh t)^{n-1}\,dt \\ &=1 \end{aligned} $$

and

15 $$\begin{aligned} &\int_{\mathbb{H}^{n}}\frac{\vert u_{k}(x) \vert ^{n}}{(d(0,x))^{\beta }}\,dV \\ &\quad =\omega_{n-1} \int_{0}^{\infty }\bigl\vert v_{k}(t) \bigr\vert ^{n}(\sinh t)^{n-1}t^{- \beta }\,dt \\ &\quad =(C_{k})^{n}\int_{0}^{e^{-k}} k^{n-1}(\sinh t)^{n-1}t^{-\beta }\,dt \\ &\quad \quad {}+(C _{k})^{n}\int_{e^{-k}}^{1} k^{-1}\vert \ln t \vert ^{n}(\sinh t)^{n-1}t^{-\beta }\,dt \\ &\quad =O\biggl(\frac{k^{n-1}}{e^{(n-\beta )k}}\biggr)+O\biggl(\frac{1}{k}\biggr) \\ &\quad =O\biggl(\frac{1}{k}\biggr). \end{aligned}$$

It follows that

16 $$\begin{aligned} &\int_{\mathbb{H}^{n}}\frac{\Phi (\alpha_{n}(1-\frac{\beta }{n})\vert u_{k} \vert ^{\frac{n}{n-1}})}{(1+u_{k})^{p}(d(0,x))^{\beta }}\,dV \\ &\quad =\omega_{n-1} \int_{0}^{\infty }\frac{\Phi (\alpha_{n}(1-\frac{\beta }{n})\vert v_{k} \vert ^{\frac{n}{n-1}})}{(1+v_{k})^{p}} t^{-\beta }( \sinh t)^{n-1}\,dt \\ &\quad \geq \frac{\Phi (\alpha_{n}(1-\frac{\beta }{n})\vert \omega_{n-1}^{-\frac{1}{n}}C_{k} k^{\frac{n-1}{n}}\vert ^{\frac{n}{n-1}})}{(1+ \vert \omega_{n-1}^{-\frac{1}{n}}C_{k} k^{\frac{n-1}{n}} \vert )^{p}} \int_{0}^{e^{-k}} (\sinh t)^{n-1}t^{-\beta }\,dt \\ &\quad \sim \frac{\Phi ((n-\beta )kC_{k}^{\frac{n}{n-1}})}{(1+\vert \omega_{n-1}^{-\frac{1}{n}}C_{k} k^{\frac{n-1}{n}} \vert )^{p}}e^{-(n-\beta )k} \\ &\quad \sim k^{-p \frac{n-1}{n}} \end{aligned}$$

as $k \rightarrow \infty $.

When $p<\frac{n}{n-1}$, we can apply (15) and (16) to obtain

17 $$\begin{aligned} &\int_{\mathbb{H}^{n}}\frac{\Phi (\alpha_{n}(1-\frac{\beta }{n})\vert u _{k} \vert ^{\frac{n}{n-1}})}{(1+u_{k})^{p}(d(0,x))^{\beta }}\,dV \biggl( \int_{\mathbb{H}^{n}}\frac{\vert u_{k}(x) \vert ^{n}}{(d(0,x))^{\alpha }}\,dV \biggr)^{-1} \\ &\quad \geq k^{1-p \frac{n-1}{n}} \\ &\quad \rightarrow \infty . \end{aligned}$$

Thus, we accomplish the proof of Theorem 2.

## Conclusions

In this paper, we prove a singular version of Moser-Trudinger inequality with the exact growth condition in the *n*-dimension hyperbolic space $\mathbb{H}^{n}$. It is well known that the Moser-Trudinger inequality plays an important role in nonlinear analysis and can be applied to study the ground state solutions of *N*-Laplacian equation with critical exponential growth. Our results represent very good progress on modern analysis and geometric inequalities.
